# Erythropoietin receptor transcription is neither elevated nor predictive of surface expression in human tumour cells

**DOI:** 10.1038/sj.bjc.6604220

**Published:** 2008-03-18

**Authors:** A M Sinclair, N Rogers, L Busse, I Archibeque, W Brown, P D Kassner, J E V Watson, G E Arnold, K C Q Nguyen, S Powers, S Elliott

**Affiliations:** 1 Amgen Inc., One Amgen Center Drive, Thousand Oaks, CA 91362, USA; 2 Cold Spring Harbor Laboratory, Cold Spring Harbor, NY 11724, USA

**Keywords:** erythropoietin receptor, expression, transcript, protein, Epo binding, tumours

## Abstract

Erythropoietin receptor (EpoR) has been reported to be overexpressed in tumours and has raised safety concerns regarding the use of erythropoiesis-stimulating agents (ESAs) to treat anaemia in cancer patients. To investigate the potential for EpoR to be overexpressed in tumours, we have evaluated human tumours for amplification of the *EPOR* locus, levels of *EPOR* transcripts, and expression of surface EpoR protein. Gene amplification analysis of 1083 solid tumours found that amplification of the *EPOR* locus was rare with frequencies similar to other non-oncogenes. *EPOR* transcript levels in tumours and tumour cell lines were low in comparison with bone marrow and were equivalent to, or lower than, levels in normal tissues of tumour origin. Although EpoR mRNA was detected in some tumour lines, no EpoR could be detected on the cell surface using ^125^I-Epo binding studies. This may be due to the lack of EpoR protein expression or lack of cell-surface-trafficking factors, such as Jak2. Taken together, we have found no evidence that EpoR is overexpressed in tumours or gets to the surface of tumour cells. This suggests that there is no selective advantage for tumours to overexpress EpoR and questions the functional relevance of EpoR gene transcription in tumours.

Erythropoiesis-stimulating agents (ESAs), epoetin-*α*, epoetin-*β*, and darbepoetin-*α*, regulate erythropoiesis by binding and activating the erythropoietin receptor (EpoR) on the surface of erythropoietic precursor cells, stimulating their survival, proliferation, and differentiation into mature red blood cells. Recently, off-label investigational clinical trials that aimed to prevent anaemia in breast and head and neck cancer patients undergoing chemotherapy and radiotherapy, respectively, reported a decrease in locoregional progression-free survival and overall survival in ESA-treated patients (Henke *et al*, 2003; Leyland-Jones *et al*, 2005). These findings have led to speculation that administration of ESAs to cancer patients may promote tumour growth through stimulating EpoR expressed on tumours. This hypothesis appeared to be supported by the literature, as some investigators have reported that tumour samples and cell lines transcribe the *EPOR* gene at high levels, that 90–100% of primary human tumours overexpress EpoR protein, and that recombinant human Epo (rHuEpo) induced proliferative, survival and migration effects on tumour cell lines (reviewed by Osterborg *et al*, 2007; Sinclair *et al*, 2007). In contrast, most clinical studies have found no increase in tumour progression or decrease in survival when ESAs are administered to cancer patients (Bohlius *et al*, 2006). Furthermore, many studies have found tumour cell lines are unresponsive to ESAs, including proliferation and survival of tumour cell lines *in vitro* (Berdel *et al*, 1991; Mundt *et al*, 1992; Rosti *et al*, 1993; Selzer *et al*, 2000; Westphal *et al*, 2002), and, of most physiological relevance, tumour growth *in vivo* (Silver and Piver, 1999; Mittelman *et al*, 2001; Stuben *et al*, 2001, 2003; Thews *et al*, 2001; Golab *et al*, 2002; Pinel *et al*, 2004; Sigounas *et al*, 2004; Ning *et al*, 2005). Thus, there is conflicting evidence for the role of EpoR in tumour growth.

Concerns have been raised about the lack of specificity of antibodies used by many studies to detect EpoR. Most studies used anti-EpoR peptide polyclonal antibodies (C-20; Santa Cruz Biotechnology, Santa Cruz, CA, USA) in immunoblotting and/or immunohistochemistry (IHC) experiments (Elliott *et al*, 2006). We and others have found that polyclonal antibodies C-20 detected a 66-kDa protein that was substantially larger than EpoR (59 kDa) and was identified to be heat-shock protein HSP70 (Elliott *et al*, 2006; Brown *et al*, 2007; Ragione *et al*, 2007). Owing to cross-reactivity with non-EpoR proteins (e.g., HSP70), C-20 was found unsuitable for IHC (Elliott *et al*, 2006; Brown *et al*, 2007). In addition, other anti-EpoR antibodies have been found to be nonspecific and insensitive and therefore inappropriate for EpoR detection (Della Ragione *et al*, 2007; Kirkeby *et al*, 2007; Laugsch *et al*, 2008; Sturiale *et al*, 2007).

Since the findings and conclusions from studies that used C-20 and other anti-EpoR antibodies to detect EpoR are inconclusive, we performed an extensive investigation of the potential for EpoR to be overexpressed in tumour cells through antibody-independent approaches.

## MATERIALS AND METHODS

### Cell lines and patient tissue samples

Human cell lines analysed were UT7/Epo (Epo-dependent megakaryoblastic leukaemia); MCF-7 (breast carcinoma); HeLa (cervical carcinoma); SHSY-5Y (neuroblastoma); CAKI-1, CAKI-2, 769P, A704, A498, SW-156, SK-NEP-1 (renal carcinomas); HT29, LS174T (colon carcinomas); and A549 (lung carcinoma). Tumour samples for representational oligonucleotide microarray analysis (ROMA) and quantitative PCR (Q-PCR) analysis were obtained from Cooperative Human Tissue Network (NCI Cancer Diagnosis Program), Duke University, University of Michigan, Asterand Inc. (Detroit, MI, USA), Cytomyx Inc. (Lexington, MA, USA), Genomics Collaborative (Cambridge, MA, USA), and as previously described (van der Horst *et al*, 2005). RNAs from paired normal and cancerous colon and lung tissue were from Ardais Corp. (Lexington, MA, USA). Other non-paired tissue mRNAs were obtained from Stratagene (La Jolla, CA, USA), Clontech (Mountainview, CA, USA), Asterand Inc., Ardais Corp., and Genpak Ltd (Brighton, UK).

### Genomic amplification

DNA for ROMA, comparative genomic hybridisation (CGH) and Q-PCR was isolated from snap-frozen tumour samples using a proteinase K method. Representational oligonucleotide microarray analysis was performed as previously described (Lucito *et al*, 2003; van der Horst *et al*, 2005), and CGH was performed as previously described (Pei *et al*, 2002). Data were normalised using a modified global loss algorithm (Smyth and Speed, 2003) (data not shown), and amplicon boundaries were determined using the GLAD algorithm (Hupe *et al*, 2004) and assembled into a database (data on file at Amgen Inc.).

**Figure 1:**
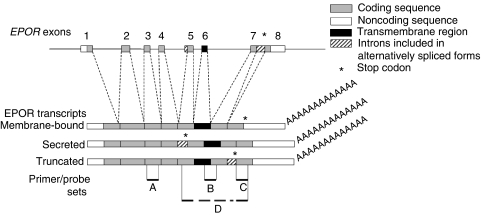
Genomic organisation of the *EPOR* locus showing alternatively spliced transcripts and location of primers and probes. Light grey boxes represent coding regions of exons 1–8, open boxes represent untranslated 5′ and 3′ regions, and black boxes represent the transmembrane coding sequences. The three major *EPOR* transcripts are shown with dashed lines representing normal splicing sites. The hatched boxes represent intronic regions contained in some alternatively spliced forms. Primer/probe sets A, B, C, and D were designed to amplify *EPOR* fragments within exons 3, 6–7, 8, and 5–8, respectively.

Quantitative PCR analysis of the *EPOR* locus was performed on DNA from 68 primary breast tumours in multiplex reactions with one of two sets of *EPOR*-specific primers and probes. Primer/probe set A amplified an *EPOR* fragment within exon 3 (Figure 1): forward primer, 5′-CTTCGTGCCCCTAGAGTTGC-3′; reverse primer, 5′-TGATGTGGATGACACGGTGAT-3′; and probe, 5′-TCACAGCAGCCTCCGGCGCT-3′. Primer/probe set C amplified an *EPOR* fragment within exon 8 that encodes the epitope for the M-20 anti-EpoR antibody (Figure 1): forward primer, 5′-TGCCAGCTTTGAGTACACTATCCT-3′; reverse primer, 5′-GCTCAGGGCACAGTGTCCAT-3′; and probe, 5′-CCCAGCTCCCAGCTCTTGCGTC-3′. Probes were labelled with 6-carboxyfluorescein (FAM) at the 5′ end and 6-carboxytetramethylrhodamine (TAMRA) at the 3′ end. A primer/probe set specific for a relatively invariant region of chromosome 6p22.2 (data on file at Amgen Inc.) was included in each multiplex reaction, as a non-amplified control: forward primer, 5′-GGTCTCTATTTGCACTTGGCTGAT-3′; reverse primer, 5′-TTTTCATTGTTGACCAAGCTAGACA-3′; and probe, 5′-TAGGGCATACTGCCTGCATATTTCCTGCT-3′ labelled with VIC at the 5′ end and TAMRA at the 3′ end. PCR products were quantified from standard curves generated with each primer/probe set using normal human genomic DNA (Novagen, Madison, WI, USA). Each 10 *μ*l Q-PCR mixture contained 5 *μ*l 2 × PCR master mix (Eurogentec, San Diego, CA, USA), 150 nM each primer and probe, and 15–20 ng genomic DNA. DNA amplification was performed using the ABI 7900 (Applied Biosystems, Foster City, CA, USA): denaturation at 95°C for 10 min, followed by 40 cycles of 15 s at 95°C and 1 min at 60°C. Reactions were performed in duplicate and repeated. Data were analysed using SDS software (Applied Biosystems). The relative copy number of *EPOR* using each primer/probe set is the ratio of the copy number at the *EPOR* locus to the copy number at the control locus; the mean relative copy number is presented.

### Quantitative RT-PCR

For laser-dissected tumour and stroma RNA, sections were cut and placed directly into extraction buffer from Picopure RNA Isolation kit (sunnythanol (2 min), Accustain (30 s), dH2O (1 min), 75% EtOH, 95% EtOH, 100% Evale; Arcturus/Molecular Devices, Sunnyvale, CA, USA) or onto glass slides, then into a slide box on dry ice. Sections were stained as follows: treated with 75% EtOH ( × 2), dipped in xylene, and then air-dried. Laser dissection was performed on a Pixcell IIe System and RNA was extracted using PicoPure Isolation kit (Arcturus/Molecular Devices), according to the manufacturer's instructions. RNA was quantified using the Beckman DU640 Spectrophotometer at the following wavelengths: 260, 280, and 320 nm.

RNA was isolated from cells and tissues using the Absolutely RNA miniprep kit (Stratagene), treated with DNase I (Roche Biochemical, Indianapolis, In, USA), and cDNA synthesised using SuperScript II (Invitrogen, Carlsbad, CA, USA). Quantitative RT-PCR was performed using three sets of *EPOR*-specific primers and probes to amplify fragments within exons 3 (primer/probe set A), 6/7 (primer/probe set B), and 8 (primer/probe set C) (Figure 1). *EPOR* was amplified from exons 3 and 8 using primer/probe sets A and C, and from exon 6/7 using primer/probe set B: forward primer, 5′-ACCGCCGGGCTCTGAA-3′; reverse primer, 5′-TTCAAACTCGCTCTCTGGGC-3′; and probe, 5′-AGAAGATCTGGCCTGGCATCCCG-3′. Human cyclophilin was amplified using forward primer, 5′-TGCTGGACCCATCACAAATG-3′; reverse primer, 5′-TGCCATCCAACCACTCAGTC-3′; and probe, 5′-TTCCCAGTTTTTCATCTGCACTGCCA-3′. Probes were labelled with FAM (5′) and TAMRA (3′). PCR mixtures contained 50 ng cDNA, TaqMan Universal PCR Master Mix (Applied Biosystems), 450 nM primers, and 200 nM probe. An RNA control was included to confirm that samples were not contaminated with genomic DNA. The amplification programme was as follows: denaturation at 95°C for 10 min, followed by 40 cycles of 95°C for 15 s and 60°C for 1 min (ABI PRISM 7700 and PRISM 7900HT Sequence Detection Systems; Applied Biosystems). Levels of *EPOR* transcripts were normalised to cyclophilin. For brain samples and head and neck laser-dissected samples, cDNA was synthesised using Qiagen's OmniScript Reverse Transcriptase kit (Qiagen, Valencia, CA, USA), using 1 *μ*M oligo dT (Invitrogen), 0.5 U *μ*l^−1^ RNase inhibitor (Roche Biochemical), 50 ng RNA, and 500 *μ*M dNTP in a 20 *μ*l reaction. Equal amount (50 ng) of the DNase-treated RNA was used as a negative RT control, and quantitative RT-PCR was performed using Fast Start SYBR Green PCR Master Mix, according to the manufacturer's instructions (Roche Biochemical), using 0.5 *μ*M of forward and reverse primers. Lightcycler 1.5 (Roche Biochemical) parameters were as follows: EpoR, 10 min at 95°C, then 45 cycles of 15 s at 95°C, 5 s at 62°C, 20 s at 72°C, 90°C for 2 s with a reading at the end of the 2 s hold time. For human cyclophilin primers, parameters were as follows: 10 min at 95°C and 45 cycles of 15 s at 95°C, 5 s at 67°C, 20 s at 72°C and 2 s at 85°C with a reading at the end of the 2 s hold time. Lightcycler Detection software (versions 4.0) was used to analyse and graph the data. Primer set D (Figure 1) was EpoR 5′-CGTATGGCTGAGCCGAGCTT-3′ (exon 5) and 5′-CAGCCATCATTCTGGTACAGC-3′ (exon 8). Human cyclophilin primers were 5′-AGACGCCACCGCCGAGGAA-3′ (exon 1) and 5′-TGCCAGGACCCGTATGCTTTAGGA-3′ (exon 4).

### Expression microarray analysis

Standard cRNA labelling and array processing were conducted according to the Affymetrix manual (Affymetrix, Santa Clara, CA, USA). First-strand synthesis used 5 *μ*g total RNA, 10 pmol T7-(dT)_24_ primer, and Superscript II (Invitrogen). Double-stranded cRNA was purified using the MinElute Reaction Cleanup kit (Qiagen). Biotinylated cRNA was synthesised using the Bioarray High Yield RNA Transcript Labeling kit (Enzo Diagnostics, Farmingdale, NY, USA) for 6 h at 37°C and purified using the RNeasy Mini kit (Qiagen). The cRNA was hybridised to the Affymetrix Human Genome U133 Plus 2.0 array containing five unique *EPOR* probe sets: 209962_AT, 209963_S_AT, 215054_AT, 37986_AT, and 396_F_AT, each identifying different subregions within *EPOR* exon 8. The arrays were washed using the EukGE_WS2v4_450 protocol on a GeneChip Fluidic Station 450 and scanned using the Affymetrix GeneChip Scanner 3000. Raw signal intensities that met the recommended quality criteria were imported into the gene expression analysis system, Rosetta Resolver 5.0 (Rosetta Bio-software, Seattle, WA, USA; http://www.rosettabio.com/tech/default.htm). System processing, including normalisation, consisted of algorithms using an error-modelling approach (Rajagopalan, 2003).

A conventional two-way analysis of variance (ANOVA) was performed to determine if *EPOR* was differentially expressed in tumour *vs* normal tissues. Normalised intensity values were log transformed to achieve homoscedacity and used for the ANOVA calculations. An ANOVA calculation was performed for each tissue type with disease state and probe set as the two factors. If a difference was found, a *post hoc* comparison using the Dunn–Sidak test was performed. Statistical calculations were made using Matlab version 6.5.1 (Mathworks Inc., Natick, MA, USA).

### Western blot analysis

Cell lysates and immunoblots were prepared as described previously (Elliott *et al*, 2006). Membranes were blocked with 5% milk in TBS containing 0.05% Tween-20, and incubated with 1 : 1000 dilution anti-Janus kinase 2 (Jak2) antibody (Cell Signaling Technologies, Danvers, MA, USA) for 2 h at room temperature. The secondary antibody was anti-rabbit IgG conjugated to horseradish peroxidase (Amersham Biosciences, Piscataway, NJ, USA). ECL-Plus (Amersham Biosciences) was used for detection. Blots were stripped and reprobed with 0.25 *μ*g ml^−1^ anti-cyclophilin B (Abcam, Cambridge, MA, USA) as a loading control.

### Epo-binding assays

Recombinant human Epo-binding studies were performed as previously described (Elliott *et al*, 1997) with some modifications. Cells were grown to confluency, dissociated with Versene (EDTA/EGTA), centrifuged, washed, and resuspended in binding buffer (RPMI, 1% BSA, 0.1% sodium azide, 50 mM HEPES, 10 *μ*g ml^−1^ cytochalasin B), and 1 × 10^6^ cells in 80 *μ*l were incubated with 133 pM ^125^I-rHuEpo (specific activity, 2500 Ci mmol^−1^), with or without a 1500-fold excess (200 nM) unlabelled rHuEpo, for 2.5 h at 37°C. Reactions were quenched with 700 *μ*l ice-cold PBS/0.5% BSA, and then centrifuged at 5000 **g**. Surface-bound ^125^I-rHuEpo was determined using a Packard Cobra II Auto Gamma Counter (Perkin-Elmer, Boston, MA, USA) and specific binding calculated as the difference in ^125^I-rHuEpo binding in the presence and absence of excess rHuEpo. For UT7/Epo Scatchard analysis, varying amounts of cold ligand (10 pg to 5 ng) in 40 *μ*l binding buffer were added to 10 *μ*l ^125^I-rHuEpo and 50 *μ*l 5 × 10^5^ UT7/Epo cells. Each reaction was performed in duplicate. Reaction tubes were incubated at 37°C for 1.5–2 h. Cell-bound and free ^125^I-rHuEpo were separated through 100% dibutyl phthalate oil in a fixed angle horizontal rotor at 12 000 **g** for 2 min at room temperature. The tubes were frozen in dry ice, the oil phase containing cell-bound ^125^I-rHuEpo was extracted, and the amount of ^125^I-rHuEpo was determined by scintillation counting. Nonspecific binding was determined by adding a 300-fold excess of cold rHuEpo to the reaction mix. Scatchard analyses were performed using Microsoft Excel.

## RESULTS

### Genomic amplification of *EPOR*

**Figure 2:**
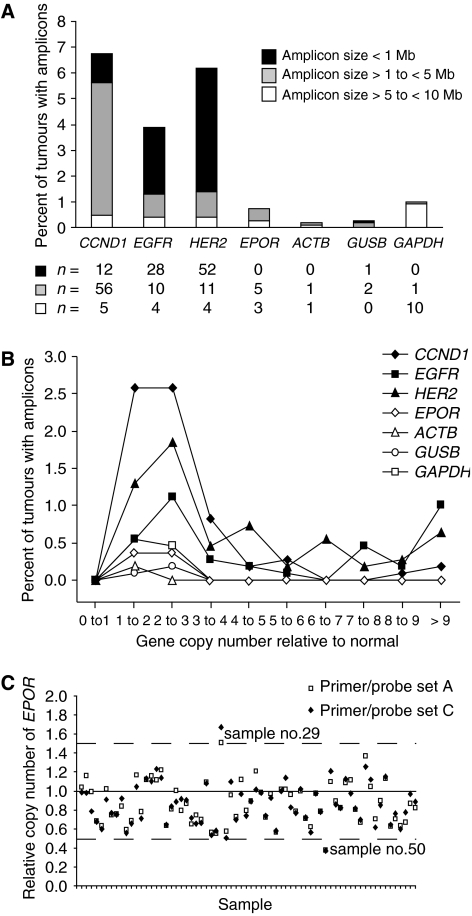
Erythropoietin receptor genomic amplification in tumour samples. Quantitative genomic microarray analysis was performed on 1083 tumours from 15 different tumour types. (**A**) Per cent of tumours demonstrating genomic amplification of oncogenes cyclin D1 (*CCND1*), *EGFR*, and *HER2*; non-oncogenes *β*-actin (*ACTB*), *GUSB*, and *GAPDH*; and test locus *EPOR*. The numbers of tumours with amplicons are shown below the *x* axis. (**B**) Per cent of tumours with genomic amplification of genes from panel A present in amplicons <10 Mb plotted against gene copy numbers. (**C**) Quantitative genomic PCR analysis of the *EPOR* locus in 68 breast tumour samples. The *EPOR*-specific primer/probe sets A and C were used to amplify *EPOR* fragments from exons 3 and 8, respectively. Breast tumour no. 29 had a gain in *EPOR* copy number (1.6-fold) and no. 50 had a deletion of one *EPOR* locus (0.4-fold).

Representational oligonucleotide microarray analysis and CGH data from 1083 tumour samples from 15 different tumour types were analysed for amplification of the *EPOR* locus. Amplification of *EPOR* in amplicons <10 Mb was identified in less than 0.7% of tumours (*n*=8) and was similar to the frequency of amplification of other established non-oncogenic loci such as glyceraldehyde-3-phosphate dehydrogenase (*GAPDH*), *β*-actin (*ACTB*), and *β*-glucuronidase (*GUSB*) (Figure 2A). None of the tumours with amplified *EPOR* contained *EPOR* in amplicons <1 Mb. Oncogenic loci cyclin D1 (*CCND1*), *HER2*, and *EGFR* were amplified in 3.5–6.7% of tumours and frequently contained amplicons <1 Mb (Figure 2A). Copy numbers of oncogenic loci were increased >9-fold in some tumours (Figure 2B). In contrast, the copy number of *EPOR* was increased to a maximum of 2–3-fold, concordant with other non-oncogenic loci (Figure 2B).

To further analyse the frequency of *EPOR* amplification, Q-PCR was performed on genomic DNA isolated from 68 primary breast tumours. Copy numbers of *EPOR* were between 0.5 and 1.5 in all tumour samples, except one tumour with a copy gain and one with a copy deletion (Figure 2C). Both *EPOR* primer sets yielded concordant results. Taken together, these results demonstrated that the *EPOR* locus was rarely amplified in primary human tumours.

### *EPOR* transcripts in tumours and tumour cell lines

**Figure 3:**
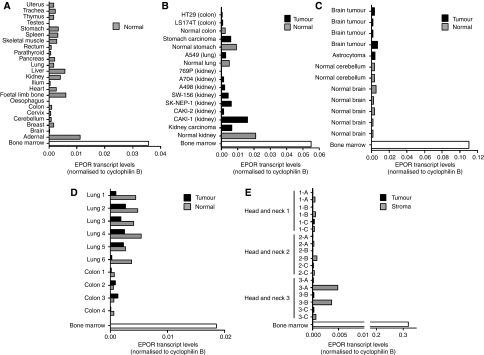
Levels of *EPOR* transcripts in normal *vs* tumour tissues. Quantitative RT-PCR was used to determine levels of *EPOR* transcripts in normal and tumour tissues relative to levels of cyclophilin B transcripts. (**A**) Levels of *EPOR* transcripts in a panel of normal tissues obtained using primer/probe set B (corroborated with primer/probe set A). *EPOR* transcript levels relative to cyclophilin were analysed: (**B**) normal tissues *vs* tumour tissues and cell lines; (**C**) brain tumours and normal brain (not patient-matched samples); (**D**) patient-matched normal *vs* colon and lung tumour samples; (**E**) patient-matched head and neck tumour and stroma (**A**–**C** indicate different preparations from the same tumour). Bone marrow samples were included as a positive haematopoietic control in all analyses (clear bars). Results obtained using primer/probe set B (Figure 1) are shown for panels A, B, and D and primer set D for panels C and E.

To investigate *EPOR* overexpression through mechanisms other than genomic amplification, levels of *EPOR* transcripts in tumours and cell lines were compared with levels in normal tissues of tumour origin. Analysis of 24 normal tissues showed that *EPOR* was transcribed at high levels in bone marrow, medium levels in adrenal, and low levels in all other tissues (Figure 3A). Comparative analysis of *EPOR* transcription in tumour *vs* non-tumour tissues determined that transcript levels in kidney, stomach, colon, and lung tumours and cell lines were lower than those found in corresponding normal tissues (Figure 3B). Levels of *EPOR* transcripts were also not elevated in brain tumours relative to normal brain (Figure 3C), colon and lung adenocarcinomas relative to patient-matched normal tissue (Figure 3D), and laser-dissected tumour epithelia from head and neck tumours relative to stroma (samples collected from three different regions from each tumour A–C, Figure 3E).

**Figure 4:**
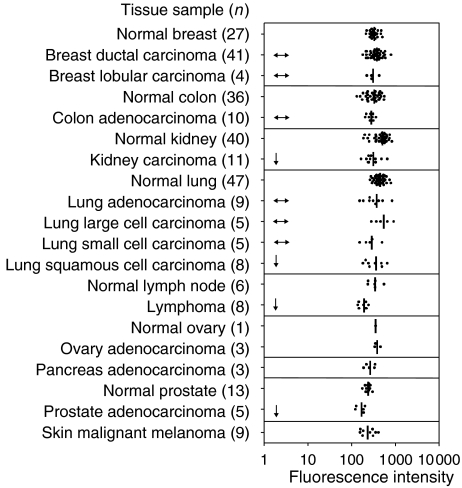
Microarray analysis of levels of *EPOR* transcripts in normal *vs* oncogenic samples. Comparative microarray analysis of 121 tumour and 170 normal tissues from breast, colon, kidney, lung, lymph node, ovary, pancreas, prostate, and skin samples. Closed circles represent transcript levels from individual samples using *EPOR* probe 396_F_AT (*EPOR* exon 8). Other *EPOR* probe sets yielded similar intensity profiles. Horizontal, double-headed arrows indicate no statistical difference in *EPOR* levels between normal and tumour tissues. A single-headed arrow indicates a significant (*P*<0.05) reduction in levels of *EPOR* transcripts in tumour tissues compared with normal tissues. No statistical analyses were performed on pancreatic samples because of the lack of a normal control, or on ovary and melanoma samples because of their small sample sizes.

Levels of *EPOR* transcripts were further examined in microarray analyses of 121 tumour and 170 normal tissues. For each of the five probe sets for *EPOR* on the HG-U133 Plus 2.0 array, levels of *EPOR* transcripts in tumours were not significantly elevated above levels found in the normal tissue counterpart (Figure 4; data not shown). Interestingly, a statistically significant (*P*<0.05) decrease in levels of *EPOR* transcripts in kidney tumours, lung squamous cell carcinoma, lymphomas, and prostate adenocarcinoma relative to normal tissues was identified (Figure 4). This result was confirmed with five different probe sets. These data demonstrate that *EPOR* transcription is not elevated in tumour samples or cell lines compared with *EPOR* transcript levels in the normal, non-tumour counterpart.

### *EPOR* transcript levels and surface expression in tumour cell lines

**Figure 5:**
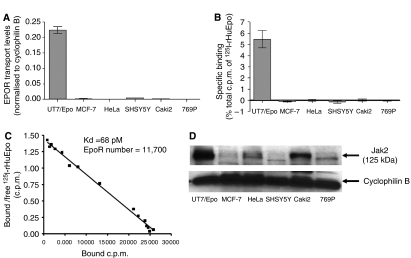
Lack of correlation between levels of *EPOR* transcripts and EpoR surface expression in tumour cell lines. (**A**) Quantitative RT-PCR of *EPOR* in tumour cell lines using primer/probe set C (Figure 1). Similar data were obtained with primer/probe sets A and B (Figure 1; data not shown). (**B**) Specific binding of ^125^I-rHuEpo to cell lines (combined data from three to four experiments with *n*=11–17 per bar). Error bars represent s.e.m. (**C**) Scatchard analysis of rHuEpo binding to UT7/Epo cells. Each point represents the average of three samples. (**D**) Western blot analysis of Jak2 expression levels. Cyclophilin B is shown as a loading control for western blot analysis.

Since *EPOR* transcripts were detected in tumour cells, albeit at low levels, we investigated the association between transcript levels and expression of EpoR at the cell surface. The particular cell lines used in this study were selected because of reports that they responded to Epo. The six cell lines included megakaryoblastic leukaemia line UT7/Epo cells (known to be Epo responsive; Laugsch *et al*, 2008) as a positive control; a renal carcinoma line 769P (with extremely low levels of EpoR mRNA) (Elliott *et al*, 2006) as a negative control; and four cell lines in which rHuEpo has been reported to induce an *in vitro* response: breast carcinoma line MCF-7 (Acs *et al*, 2001), cervical carcinoma line HeLa (Acs *et al*, 2003; Pajonk *et al*, 2004), renal carcinoma line CAKI-2 (Westenfelder and Baranowski, 2000), and neuroblastoma line SHSY-5Y (Um *et al*, 2007). Relatively high levels of *EPOR* transcripts were observed in UT7/Epo cells, very low levels in MCF-7, HeLa, SHSY-5Y, and CAKI-2 cells, and negligible levels in 769P cells (Figure 5A). While UT7/Epo cells bound high levels, none of the solid tumour cell lines bound detectable levels above background of ^125^I-rHuEpo, (Figure 5B). Scatchard analysis demonstrated that UT7/Epo cells expressed ∼11 700 cell-surface receptors per cell with a binding affinity of 68 pM (Figure 5C). In contrast, 32D cells expressing murine EpoR expressed relatively low levels of cell-surface receptors (0.58±0.10% total c.p.m.; ∼760 receptors per cell; data not shown), demonstrating the assay was sensitive. These data indicate that synthesis of EpoR mRNA in solid tumour cell lines does not predict surface expression; EpoR protein may not be synthesised in these solid tumour cell lines and/or may be trapped in the cytoplasm and may not be trafficked to the surface of the cell.

Janus kinase 2 is required for surface expression of EpoR in haematopoietic cells (Huang *et al*, 2001). Variable levels of Jak2 expression were observed in cell lines (Figure 5D), and lack of Jak2 in some may account for, in part, lack of surface EpoR. However, other cell lines (e.g., CAKI-2) expressed Jak2 protein but still no surface expression was observed (Figure 5B). These data suggest that if EpoR protein is synthesised in these cells, it does not get to the surface of tumour cell lines at detectable levels due to potential limiting surface-trafficking factors, such as Jak2.

## DISCUSSION

Although ESAs have been used safely for numerous years, results from several recent off-label, investigational clinical trials have raised concerns that ESAs may have direct tumour-stimulating effects and promote tumour progression in anaemic cancer patients (Henke *et al*, 2003; Leyland-Jones *et al*, 2005; Bohlius *et al*, 2006). The notion that EpoR was overexpressed in tumours and that rHuEpo enhanced tumour progression has been confounded by the finding that antibodies used to detect EpoR are nonspecific and additionally bind non-EpoR proteins, including HSP70 (Elliott *et al*, 2006; Brown *et al*, 2007; Della Ragione *et al*, 2007; Kirkeby *et al*, 2007; Laugsch *et al*, 2008; Ragione *et al*, 2007; Sturiale *et al*, 2007). Therefore, a recent study suggesting high-level EpoR expression in head and neck tumours correlated with tumour progression and worse survival in patients administered ESAs (Henke *et al*, 2006) most likely identified the well-know association of HSP70 and worse prognosis (Schmitt *et al*, 2007). Furthermore, there are a large number of studies that find that ESAs do not stimulate tumour progression in preclinical models (reviewed by Osterborg *et al*, 2007; Sinclair *et al*, 2007). Since specific anti-EpoR antibodies have yet to be identified, we investigated if EpoR was overexpressed in tumours by performing a systematic analysis of *EPOR* genomic amplification and transcription in more than 15 different primary tumour types, and EpoR transcript and surface EpoR expression analysis in representative tumour cell lines in which rHuEpo has been reported to induce responses (Westenfelder and Baranowski, 2000; Acs *et al*, 2001, 2003; Pajonk *et al*, 2004; Um *et al*, 2007).

A common phenomenon of tumour formation is the amplification of proto-oncogenes such as *HER2* (Parkes *et al*, 1990), *EGFR* (Reissmann *et al*, 1999), *CCND1* (Szepetowski *et al*, 1992; Reissmann *et al*, 1999), and *c-MET* (Rege-Cambrin *et al*, 1992), which provide a selective advantage for tumour cell growth and survival through overexpression. Genomic analysis of 1083 tumours showed that the *EPOR* locus was amplified in <0.7% tumours, only in large amplicons, and less than 2–3 times the normal copy number. In contrast, *EGFR*, *CCND1*, and *HER2* were amplified in 3.5–6.7% of tumours, often >9 times the normal copy number and frequently amplified in small amplicons indicative of the selective amplification of these genes. These data demonstrate that *EPOR* amplification is a rare event in solid tumours and not a primary driver of tumour formation and progression.

Although the *EPOR* locus was rarely amplified in tumours, it was possible that *EPOR* was overexpressed through other mechanisms. Here, we report the first comprehensive, quantitative analysis of *EPOR* transcript levels in multiple tumour types compared to tissues of tumour origin. In this analysis, we found that levels of *EPOR* transcripts in tumour samples and cell lines from more than 15 different tumour types were equivalent to, or lower than, levels in normal tissues. In concordance with our study, it was recently reported that levels of *EPOR* transcripts in prostate (Feldman *et al*, 2006) and head and neck tumours (Winter *et al*, 2005) were similar to levels in normal tissues. High levels of *EPOR* transcripts were reported in kidney tumours compared with normal kidney (Lee *et al*, 2005) and in melanoma cell lines compared with normal melanocytes (Selzer *et al*, 2000). However, these latter studies used small numbers of samples, and levels of transcripts were not quantified. In the present study, we examined 11 kidney and 9 melanoma tumour samples and found no increase in levels of *EPOR* transcripts relative to normal tissues.

Most solid tumour cell lines we examined expressed EpoR mRNA; however, the levels were substantially lower compared to that found in Epo-responsive cells (UT7/Epo) or tissues (bone marrow). In addition, we were unable to detect EpoR on the cell surface of the same cells. Owing to non-specificities and insensitivity of antibodies available for EpoR protein detection (Elliott *et al*, 2006; Brown *et al*, 2007; Della Ragione *et al*, 2007; Kirkeby *et al*, 2007; Laugsch *et al*, 2008; Sturiale *et al*, 2007), we have been unable to determine EpoR protein expression in these lines. However, the lack of detectable surface binding of ^125^I-Epo to intact cells suggests that EpoR protein levels may be low if expressed at all. Translocation of EpoR to the cell surface is an inefficient process (less than 1% of total cellular EpoR molecules are produced on the cell surface); thus, *EPOR* transcription and EpoR protein synthesis does not always lead to cell-surface expression of functional EpoR in tumour cells (Migliaccio *et al*, 1991; Neumann *et al*, 1993; Sawyer and Hankins, 1993; Hilton *et al*, 1995; Hermine *et al*, 1996; Kurten *et al*, 1996; Supino-Rosin *et al*, 1999). Thus, an alternative explanation may be inefficient transport of EpoR protein to the cell surface. Janus kinase 2 is essential for surface expression of EpoR; in haematopoietic cells, it binds EpoR in the endoplasmic reticulum, induces correct protein folding, and localises EpoR to the cell surface (Huang *et al*, 2001). Some of the cell lines investigated had no or very low Jak2 expression and may, in part, account for the lack of detectable EpoR on the surface in some cells if protein is synthesised. Interestingly, murine 32D-Epo-independent cells expressed Jak2 (DaSilva *et al*, 1994) and EpoR, but EpoR was not detected at the cell surface (Migliaccio *et al*, 1991). This suggests that accessory proteins other than Jak2 may also be required to modulate surface expression of EpoR or may alter the affinity of EpoR for Epo (Dong and Goldwasser, 1993; Masuda *et al*, 1993; Nagao *et al*, 1993; Hermine *et al*, 1994). Recently, it has been hypothesised that an alternative receptor for rHuEpo, a heterodimer of EpoR and the *β*-common chain, mediates tissue-protective effects in non-haematopoietic tissues (Brines *et al*, 2004). However, these data were not reproduced by other investigators (Um *et al*, 2007). Taken together, these data suggest that Epo responses in these cells, if any, may be weak.

It is possible that very low levels of surface receptor on the tumour cell lines we examined are present and they mediate a response to rHuEpo, thereby explaining the responses to rHuEpo reportedly detected by some investigators (Westenfelder and Baranowski, 2000; Acs *et al*, 2001, 2003; Um *et al*, 2007). Differentiated neuroblastoma cell line SHSY-5Y was reported to express extremely low (∼17) EpoR homodimers on the cell surface (Um *et al*, 2007). This level of surface receptor was reportedly sufficient to induce low-level signalling and survival responses (Um *et al*, 2007). A proliferative effect of rHuEpo was reported in MCF-7 cells (Acs *et al*, 2001), but seven other studies reported no proliferative effect in MCF-7 cells (Berdel *et al*, 1991, 1992; Mundt *et al*, 1992; Rosti *et al*, 1993; Gewirtz *et al*, 2006; Li *et al*, 2006; Laugsch *et al*, 2008). Also, rHuEpo reportedly increased the survival of parental HeLa cells in one study (Acs *et al*, 2003) but not in another study (Pajonk *et al*, 2004). Renal carcinoma cell line CAKI-2 was reported to bind ^125^I-Epo and proliferate *in vitro* a maximum of 2.5-fold (Westenfelder and Baranowski, 2000). Since most of these studies had technical concerns (e.g., performed in the absence of serum, lack of critical controls (e.g., vehicle to control for contaminating carrier proteins), use of suprapharmacologic doses of rHuEpo, modest proliferative responses for established cell lines), the results from these studies are inconclusive and inconsistent. In one such study, the direct effect of rHuEpo to induce signalling and protect a rat mammary carcinoma cell line from Taxol-induced apoptosis *in vitro* did not translate to an effect on Taxol-induced tumour inhibition *in vivo*, despite a reported impact on signalling pathways in the tumour (Hardee *et al*, 2006). The conclusion that ESAs promote tumour cell growth is not supported by *in vivo* studies in rodent tumour models: in 23 published studies, there was no tumour-promoting effect of ESAs either alone or in combination with radiotherapy or chemotherapy (Sinclair *et al*, 2007). In some studies, ESAs were reported to enhance the efficiency of tumour-ablative therapy (Silver and Piver, 1999; Mittelman *et al*, 2001; Thews *et al*, 2001; Golab *et al*, 2002; Pinel *et al*, 2004; Sigounas *et al*, 2004; Ning *et al*, 2005) and induce tumour regression (Mittelman *et al*, 2001). Therefore, the most physiologically relevant tumour models that directly examined effects of ESAs *in vivo* do not support a tumour-promoting effect of ESAs.

In summary, we found no evidence that EpoR mRNA was overexpressed in the solid tumour types analysed in this study (through genomic amplification or other mechanisms). Erythropoietin receptor transcript levels were low and EpoR protein was not trafficked to the cell surface at detectable levels. These data suggest that primary tumour samples and tumour cell lines did not overexpress EpoR. Taken together, these results question the hypothesis that there is functional relevance to EpoR mRNA transcription in tumours.
